# Supplementary material for: Chromothripsis during telomere crisis is independent of NHEJ, and consistent with a replicative origin
Source: Genome Res. 2019 May;29(5):737–49. doi: 10.1101/gr.240705.118 (PMC6499312; doi:10.1101/gr.240705.118)
Supplement: Supplemental Material [file supp_gr.240705.118_Supplemental_file_1.zip › contigs/annotated_contigs/DB93/contig.2.DB93_length_496_mean_cov_7.25806451613.docx]

**DB93_length_496_mean_cov_7.25806451613**

```
TTGGATAACTTTGCCAATAACTAGAACTCCCTCAAAAACAAAGGCTAGGTCCCTGACTCCTATAGGCATGACCAAGAGTATACTACAAA
 >chr8:17757800-17757997 + E=6e-107
ATGATTCCTCTGAGACTGAAATGTTTTTTTCCTGCTTCCCCCTCAGAGACACATTGTGGAAAAATTCTAGAACTTTTAGAATAGACATT

GTGTGCTTGACTCCAGCGA|GCTGCTTTGGCCTGCCGGTATATCTTTGTTCGGAAAGGCCCTGTCATTCCTAATTATTAATTACTAGAA
                     >chr8:17782449-17782748 + E=2e-168
TGGTTTATGCAGTACCGTTTGAAATTTATTTGTCTTCACAGAATTTTTTCAACCTGTTTGAATTTGTCCTATAACTGGATAGCCTCTCT

TTCCAAATCCATTTAATCTCAAGTTCTCTCCTACAACATTCCTATTAATTGGCTGTTCATGAACTCTACTTGTCGAACTTGTTATACCT

TGACCGAAATTTAGTTGTCTAAAATTACTTTATTATTATTTTTTGTACTTCG
```
